# Supplementary material for: Rare earth element geochemistry of Middle Devonian reefal limestones of the Dianqiangui Basin, South China: implications for nutrient sources and expansion of the reef ecosystem
Source: PeerJ. 2022 Jul 22;10:e13663. doi: 10.7717/peerj.13663 (PMC9310798; doi:10.7717/peerj.13663)

[29.raw]	S/M Hit Listing

SCAN: 5.0/140.0/0.02/8.888888E-02(sec), Cu(40kV,40mA), I(max)=40353, 02/28/22 14:38

NOTE: Intensity = Counts, 2T(0)=0.0(deg), S/M: Default Search_Match
J-Column: [+] Common/Good Patterns, [?] Uncommon/Non-Ambient Patterns, [ ] Intermediate Patterns, [D] Deleted
D-Column: C=Calculated, D=Diffractometer, F=Densitometer, V=Film/Visual, X=Other/Unknown

#	1 Hits Sorted on Figure-Of-Merit	FOM	I%	2T(0)	d/d(0)	PDF-#	J	D	#d/I
1	☐ Quartz, syn - SiO_2	1.0	64	0.120	1.000	46-1045	+	D	55

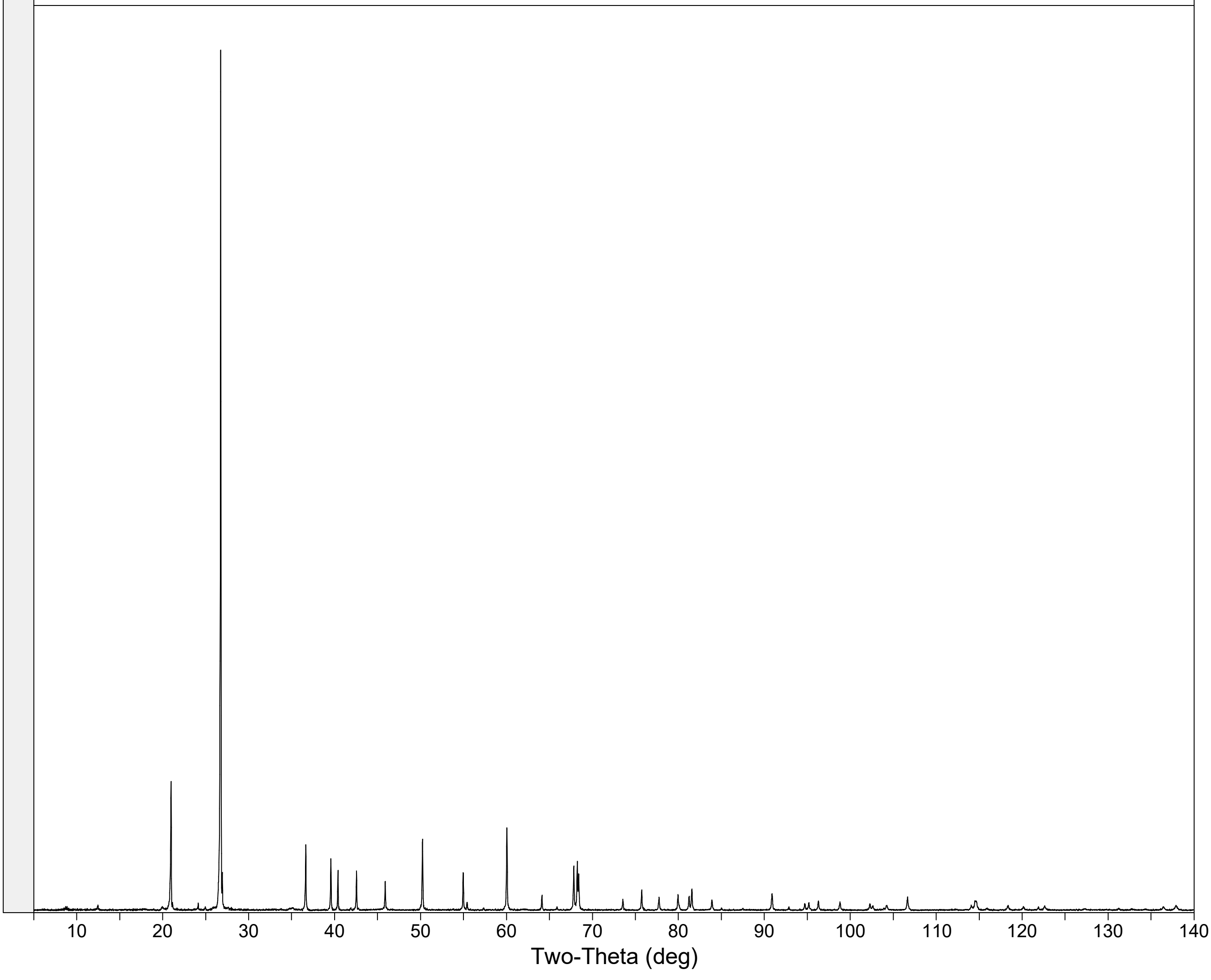

Supplement: Supplemental Information 3 [file peerj-10-13663-s003.zip › XRD Data/BZ-1.pdf]
