# Supplementary material for: Rare earth element geochemistry of Middle Devonian reefal limestones of the Dianqiangui Basin, South China: implications for nutrient sources and expansion of the reef ecosystem
Source: PeerJ. 2022 Jul 22;10:e13663. doi: 10.7717/peerj.13663 (PMC9310798; doi:10.7717/peerj.13663)

[50.raw]	S/M Hit Listing

SCAN: 5.0/140.0/0.02/8.888888E-02(sec), Cu(40kV,40mA), I(max)=3607, 03/01/22 16:30

NOTE: Intensity = Counts, 2T(0)=0.0(deg), S/M: Default Search_Match
J-Column: [+] Common/Good Patterns, [?] Uncommon/Non-Ambient Patterns, [ ] Intermediate Patterns, [D] Deleted
D-Column: C=Calculated, D=Diffractometer, F=Densitometer, V=Film/Visual, X=Other/Unknown

#	40 Hits Sorted on Figure-Of-Merit	FOM	I%	2T(0)	d/d(0)	PDF-#	J	D	#d/I
1	☐ Dolomite - CaMg(CO3)2	2.3	88	-0.040	1.000	36-0426	+	D	41
2	☐ Ankerite - Ca(Fe+2,Mg)(CO3)2	4.7	98	0.040	1.000	41-0586	+	D	41
3	☐ Quartz, syn - SiO2	5.7	81	0.060	1.000	46-1045	+	D	55
4	☐ Minrecordite - CaZn(CO3)2	12.2	89	-0.060	1.000	35-0667	+	D	20
5	☐ Halloysite-10A - Al2Si2O5(OH)4!2H2O	13.8	5	-0.040	1.000	29-1489	+	D	7
6	☐ Muscovite-3T - (K,Na)(Al,Mg,Fe)2(Si3.1Al0.9)O10...	15.0	5	0.100	1.000	07-0042	+	D	28
7	☐ Muscovite-1M, syn - KAl2Si3AlO10(OH)2	16.2	5	0.080	1.000	07-0025	+	D	29
8	☐ Phlogopite-1M - KMg3(Si3Al)O10(OH)2	18.1	5	-0.060	1.000	10-0495	+	D	26
9	☐ Muscovite-2M1, ammonian - (K,NH4,Na)Al2(Si,Al...	22.0	5	0.120	1.000	46-1311		D	22
10	☐ Glauconite-1M [NR] - K(Fe,Al)2(Si,Al)4O10(OH)2	22.9	5	0.060	1.000	09-0439	+	V	23
11	☐ Kutnohorite, magnesian - Ca(Mn,Mg)(CO3)2	23.0	70	0.100	1.000	43-0695	+	D	39
12	☐ Annite-1M, aluminian - K2(Fe5+2Al)Si5Al3O20(O...	24.3	5	0.040	1.000	26-0909	+	D	14
13	☐ Phlogopite-1M, ferroan - K(Mg,Fe)3(Al,Fe)Si3O10...	26.8	5	0.040	1.000	42-1437	+	D	33
14	☐ Lanarkite, syn - Pb2(SO4)O	27.4	27	-0.020	1.000	37-0516	+	C	49
15	☐ Gismondine - CaAl2Si2O8!4H2O	27.6	81	0.020	1.000	20-0452	+	F	64
16	☐ Bonshtedtite - Na3Fe+2(PO4)(CO3)	27.9	33	-0.100	1.000	35-0678	+	D	36
17	☐ Fluorannite - KFe3AlSi3O10F2	28.3	7	0.100	1.000	53-1188		D	13
18	☐ Phlogopite-2M1 - KMg3(Si3Al)O10(OH)2	28.8	5	0.080	1.000	10-0493	+	D	30
19	☐ Illite-2M1 [NR] - (K,H3O)Al2Si3AlO10(OH)2	28.9	18	-0.060	1.000	26-0911	+	D	18
20	☐ Phlogopite-3T - KMg3(Si3Al)O10(OH)2	31.0	5	0.040	1.000	10-0492	+	D	26
21	☐ Iimoriite-(Y) - Y2(SiO4)(CO3)	31.5	81	-0.060	1.000	35-0640	+	D	59
22	☐ Annite-1M - KFe3+2(Si,Al)4O10(OH)2	31.7	5	-0.080	1.000	42-1413	+	D	43
23	☐ Tobelite 1M, syn - NH4Al3Si3O10(OH)2	32.1	3	0.080	1.000	53-1182	+	D	50
24	☐ Ferroceladonite - K2Fe2+2Fe2+3Si8O20(OH)4	32.4	5	-0.040	1.000	54-0782	+	V	42
25	☐ Brushite, syn - CaPO3(OH)!2H2O	33.0	3	0.000	1.000	09-0077	+	X	39
26	☐ Tobelite-1M, potassian - [(NH4),K](Si,Al)4Al2O10...	33.1	3	0.080	1.000	46-1344	+	D	24
27	☐ Darapskite, syn - Na3(NO3)(SO4)!H2O	33.1	3	0.080	1.000	23-1408	+	X	52
28	☐ Muscovite-2M1 - KAl2(Si3Al)O10(OH,F)2	33.4	33	-0.080	1.000	06-0263	+	D	78
29	☐ Earlshannonite - MnFe2(PO4)2(OH)2!4H2O	33.7	7	-0.060	1.000	38-0364	+	X	41
30	☐ Wickenburgite - CaPb3Al2Si10O24(OH)6	33.7	5	0.060	1.000	21-0148	+	X	48
31	☐ Paragonite-2M1, K-deficient - NaAl2(Si,Al)4O10(O...	34.1	7	-0.100	1.000	27-0020		X	33
32	☐ Oyelite - Ca10B2Si8O29!12H2O	34.3	30	0.000	1.000	41-1386	+	D	17
33	☐ Polylithionite-1M, ferroan - K(Li,Al,Fe)3Si4O10(F,...	34.4	3	0.100	1.000	14-0565		X	30
34	☐ Pseudomalachite - Cu5(PO4)2(OH)4	35.4	5	0.100	1.000	36-0408	+	D	94
35	☐ Calciobetafite - Ca2(Ti,Nb)2O7	37.2	3	0.040	1.000	42-0002	+	C	11
36	☐ Boulangerite - Pb5Sb4S11	37.2	3	0.080	1.000	18-0688	+	D	39
37	☐ Gordonite - MgAl2(PO4)2(OH)2!8H2O	39.3	7	-0.100	1.000	14-0313	+	X	38
38	☐ Moganite - SiO2	39.4	58	-0.040	1.000	52-1425	+	X	29
39	☐ Sidorenkite - Na3Mn(PO4)(CO3)	39.6	27	0.120	1.000	33-1266	+	D	39
40	☐ Whitmoreite - FeFe2(PO4)2(OH)2!4H2O	40.3	5	0.060	1.000	26-1138	+	V	37

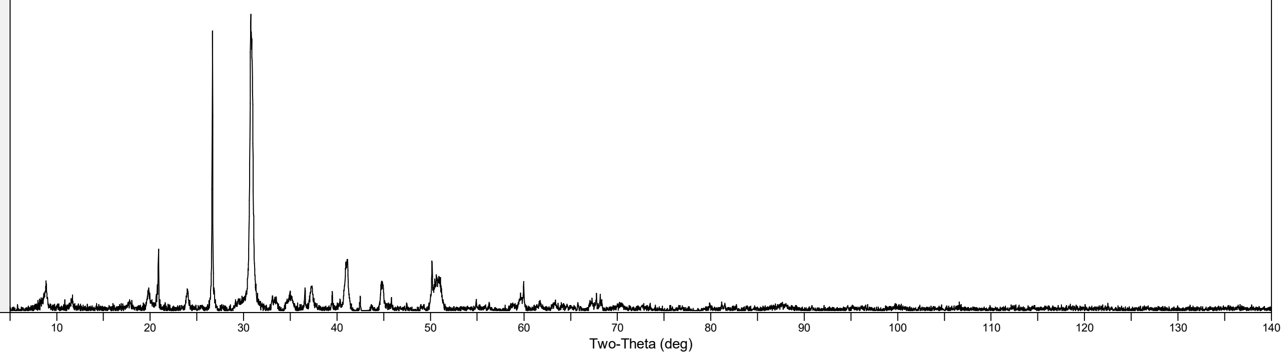

Supplement: Supplemental Information 3 [file peerj-10-13663-s003.zip › XRD Data/JWZ-2.pdf]
