# Supplementary material for: Rare earth element geochemistry of Middle Devonian reefal limestones of the Dianqiangui Basin, South China: implications for nutrient sources and expansion of the reef ecosystem
Source: PeerJ. 2022 Jul 22;10:e13663. doi: 10.7717/peerj.13663 (PMC9310798; doi:10.7717/peerj.13663)

[53.raw]	S/M Hit Listing

SCAN: 5.0/140.0/0.02/8.888888E-02(sec), Cu(40kV,40mA), I(max)=5885, 03/01/22 17:06

NOTE: Intensity = Counts, 2T(0)=0.0(deg), S/M: Default Search_Match
J-Column: [+] Common/Good Patterns, [?] Uncommon/Non-Ambient Patterns, [ ] Intermediate Patterns, [D] Deleted
D-Column: C=Calculated, D=Diffractometer, F=Densitometer, V=Film/Visual, X=Other/Unknown

#	7 Hits Sorted on Figure-Of-Merit	FOM	I%	2T(0)	d/d(0)	PDF-#	J	D	#d/I
1	☐ Calcite, syn - CaCO3	1.7	56	0.100	1.000	05-0586	+	D	45
2	☐ Quartz, syn - SiO2	23.4	12	-0.020	1.000	46-1045	+	D	55
3	☐ Briartite, syn - Cu2FeGeS4	25.9	30	0.100	1.000	42-0565	+	C	43
4	☐ Rosenbuschite - (Ca,Na)6ZrTi(Si2O7)2F2(F,OH)	28.1	34	0.100	1.000	51-1572	+	D	64
5	☐ Renierite - Cu10Fe4ZnGe2S16	32.6	30	0.100	1.000	41-0591	+	D	24
6	☐ Fersmite, syn - CaNb2O6	40.6	34	0.100	1.000	39-1392	+	D	69
7	☐ Nagashimalite - Ba4(V+3,Ti)4ClSi8B2O27(O,OH)2	42.8	44	0.080	1.000	33-0188	+	D	77

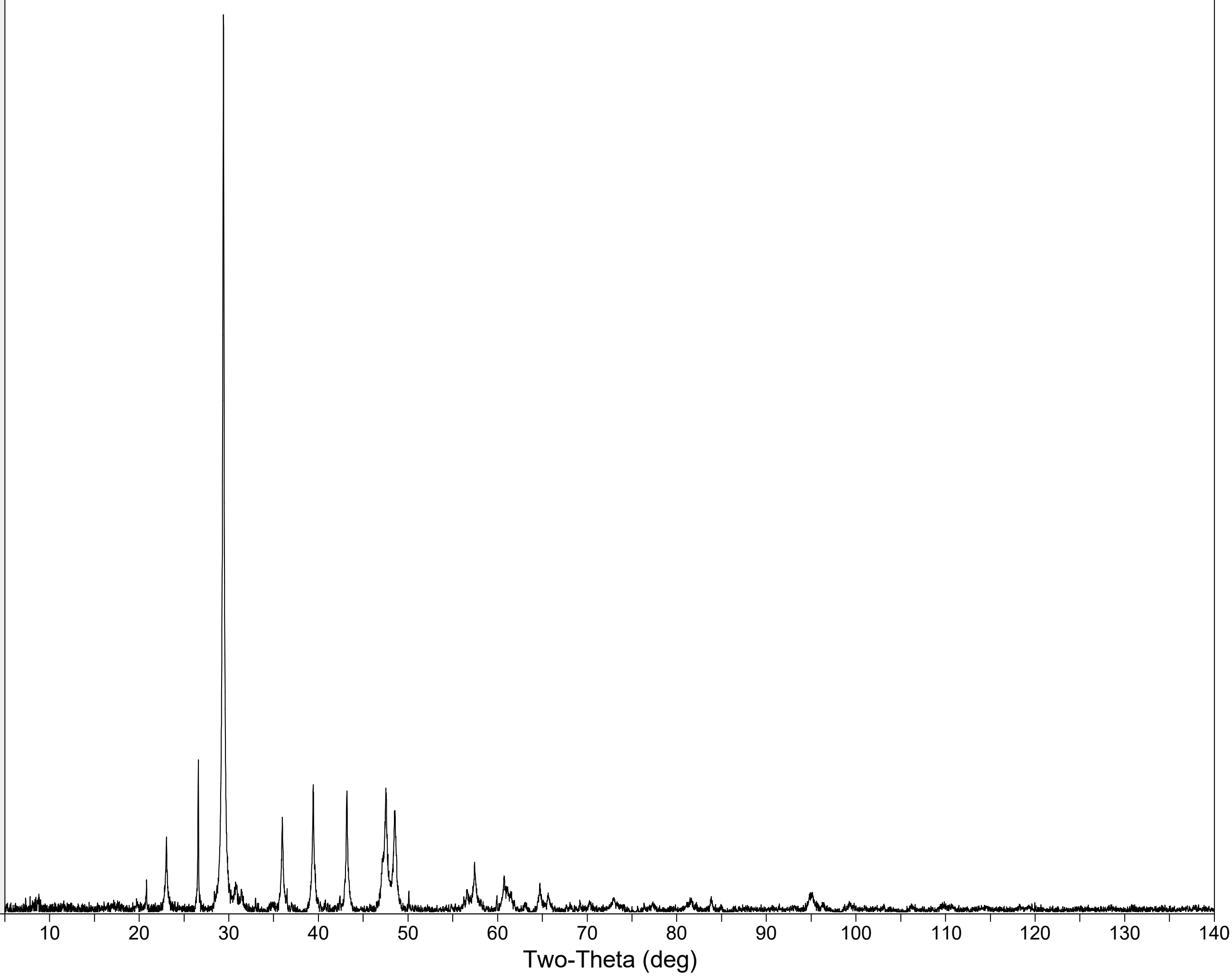

Supplement: Supplemental Information 3 [file peerj-10-13663-s003.zip › XRD Data/JWZ-5.pdf]
