# Supplementary figures and images for: Rare earth element geochemistry of Middle Devonian reefal limestones of the Dianqiangui Basin, South China: implications for nutrient sources and expansion of the reef ecosystem
Source: PeerJ. 2022 Jul 22;10:e13663. doi: 10.7717/peerj.13663 (PMC9310798; doi:10.7717/peerj.13663)

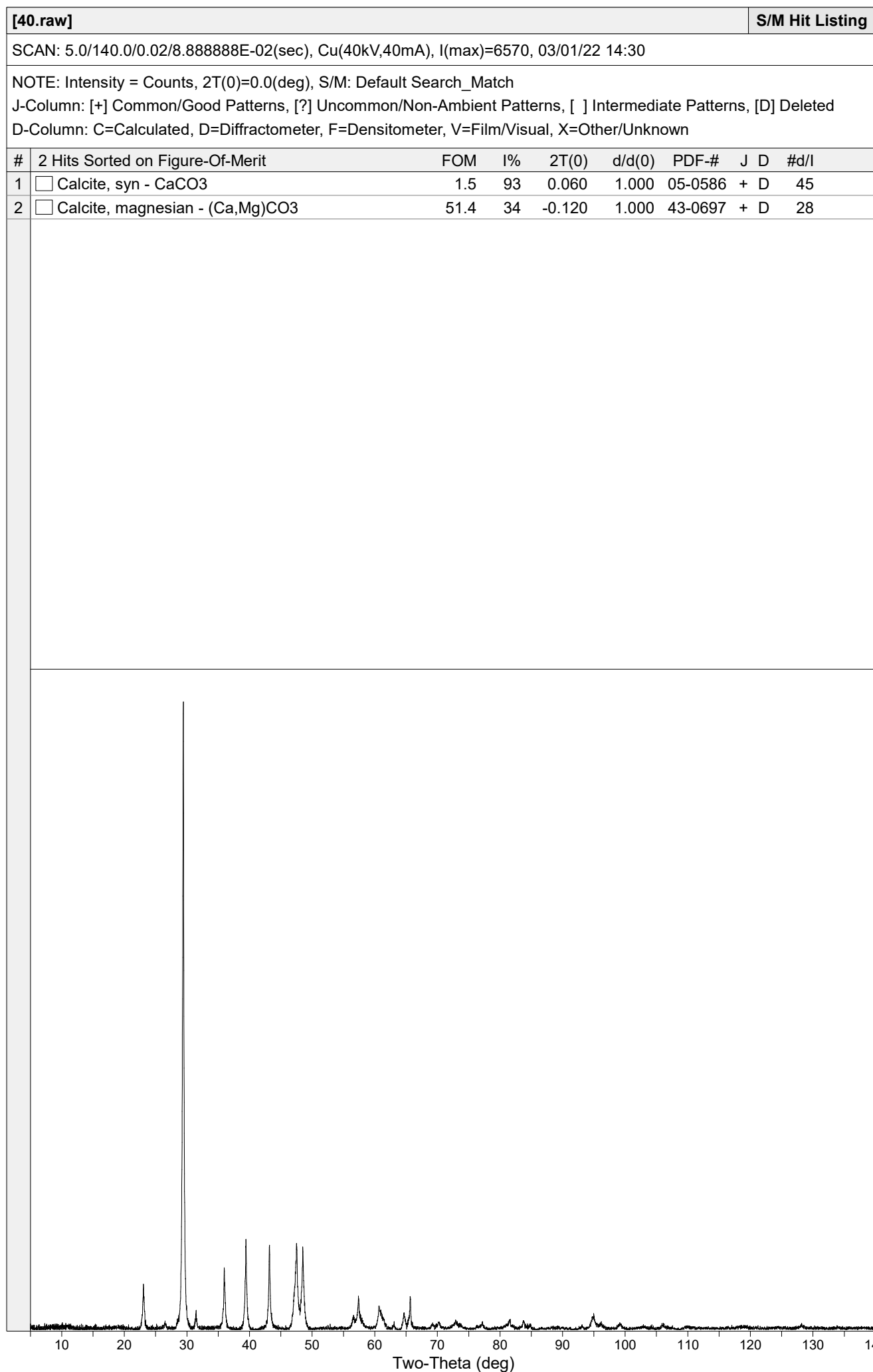

Supplement: Supplemental Information 3 [file peerj-10-13663-s003.zip › XRD Data/BZ-12.pdf]

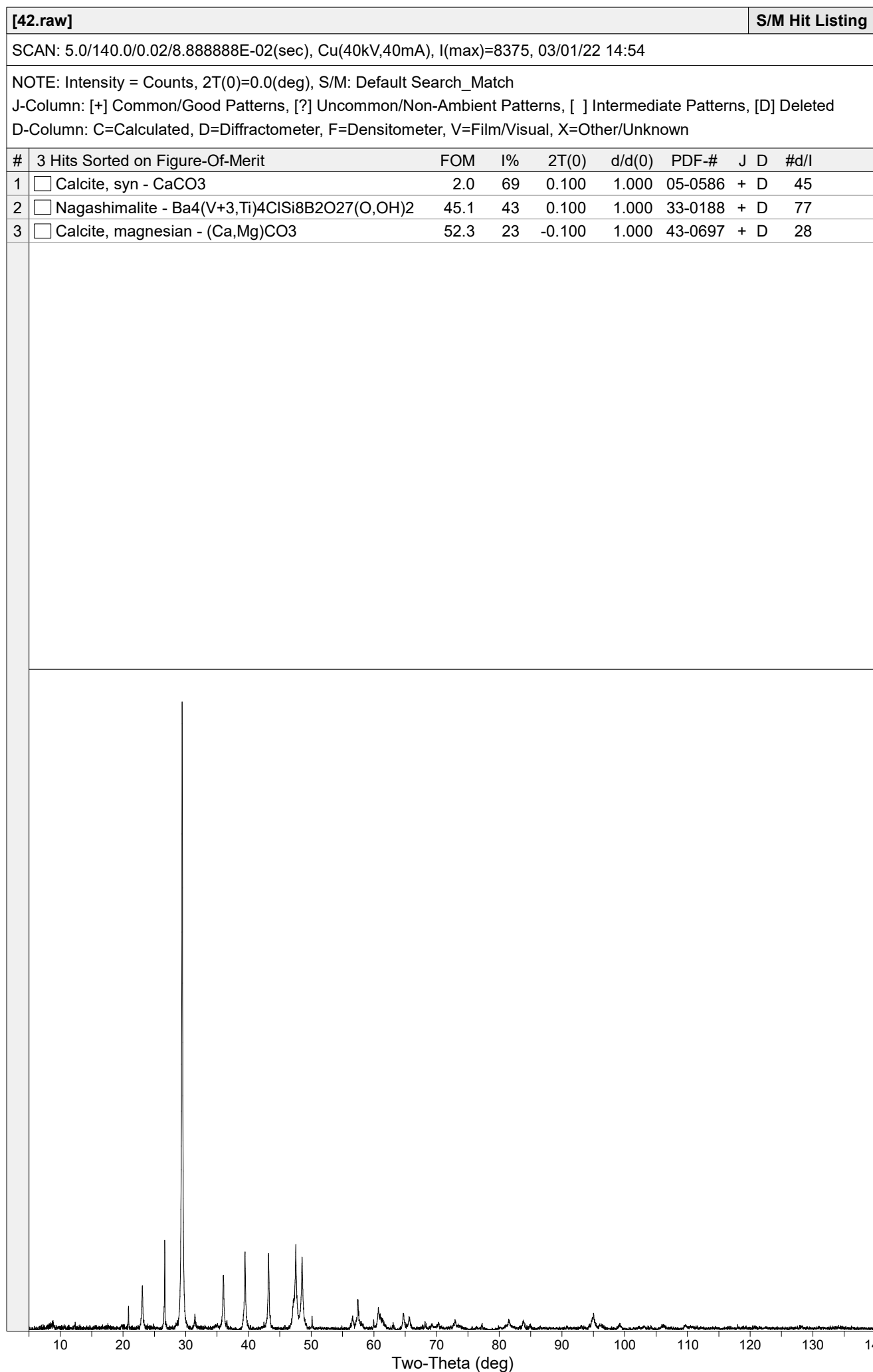

Supplement: Supplemental Information 3 [file peerj-10-13663-s003.zip › XRD Data/BZ-14.pdf]

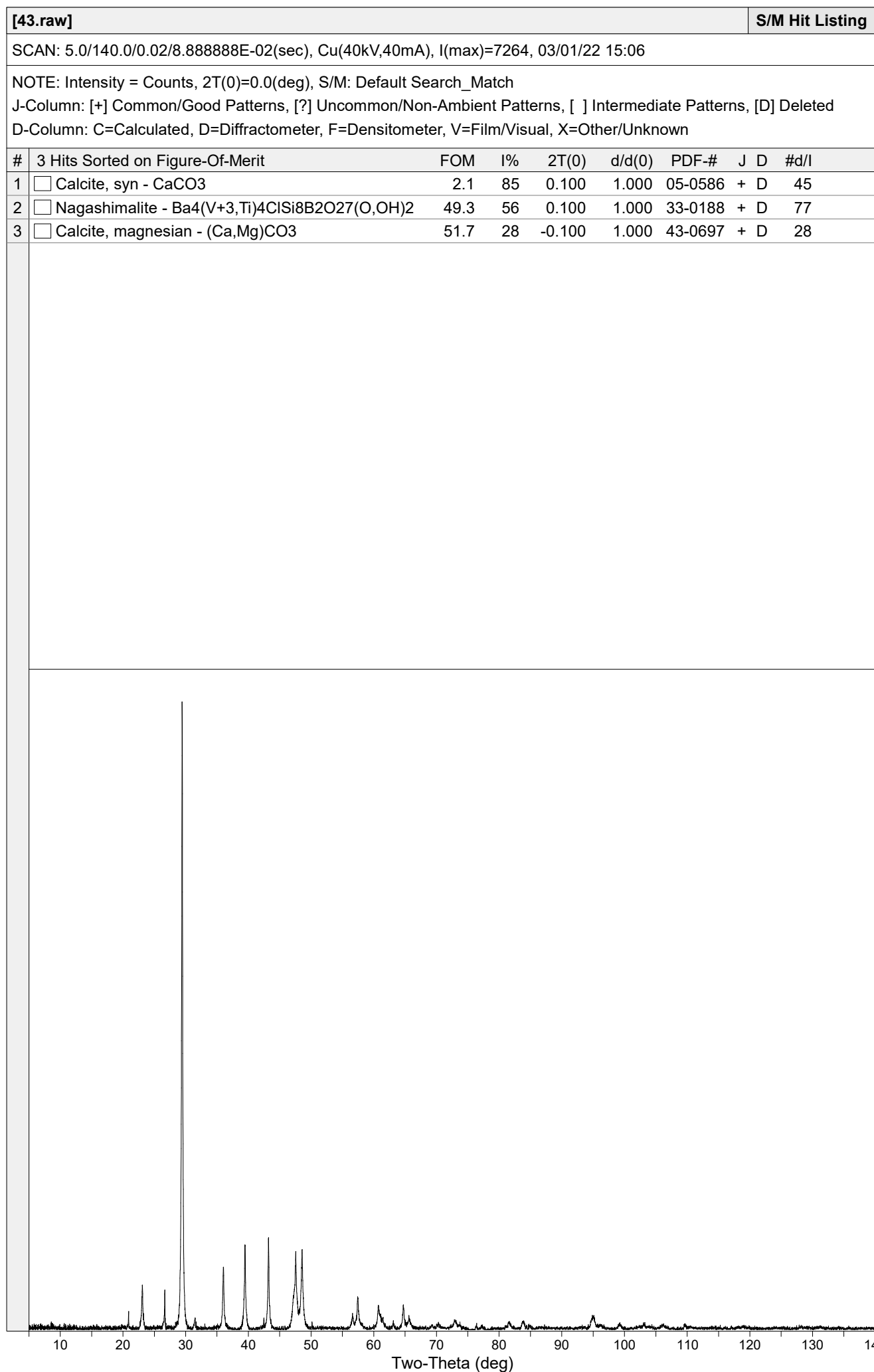

Supplement: Supplemental Information 3 [file peerj-10-13663-s003.zip › XRD Data/BZ-15.pdf]

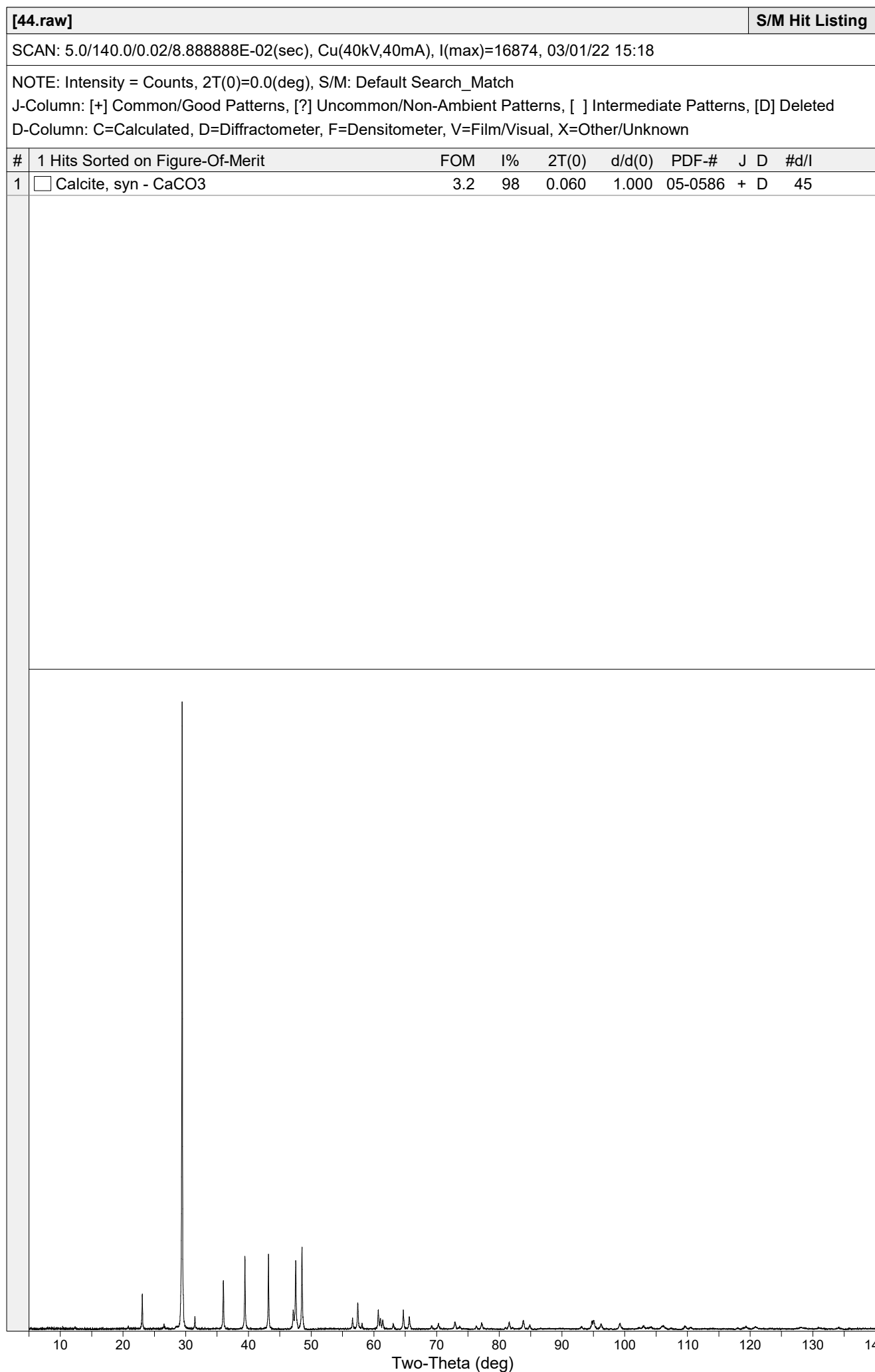

Supplement: Supplemental Information 3 [file peerj-10-13663-s003.zip › XRD Data/BZ-16.pdf]

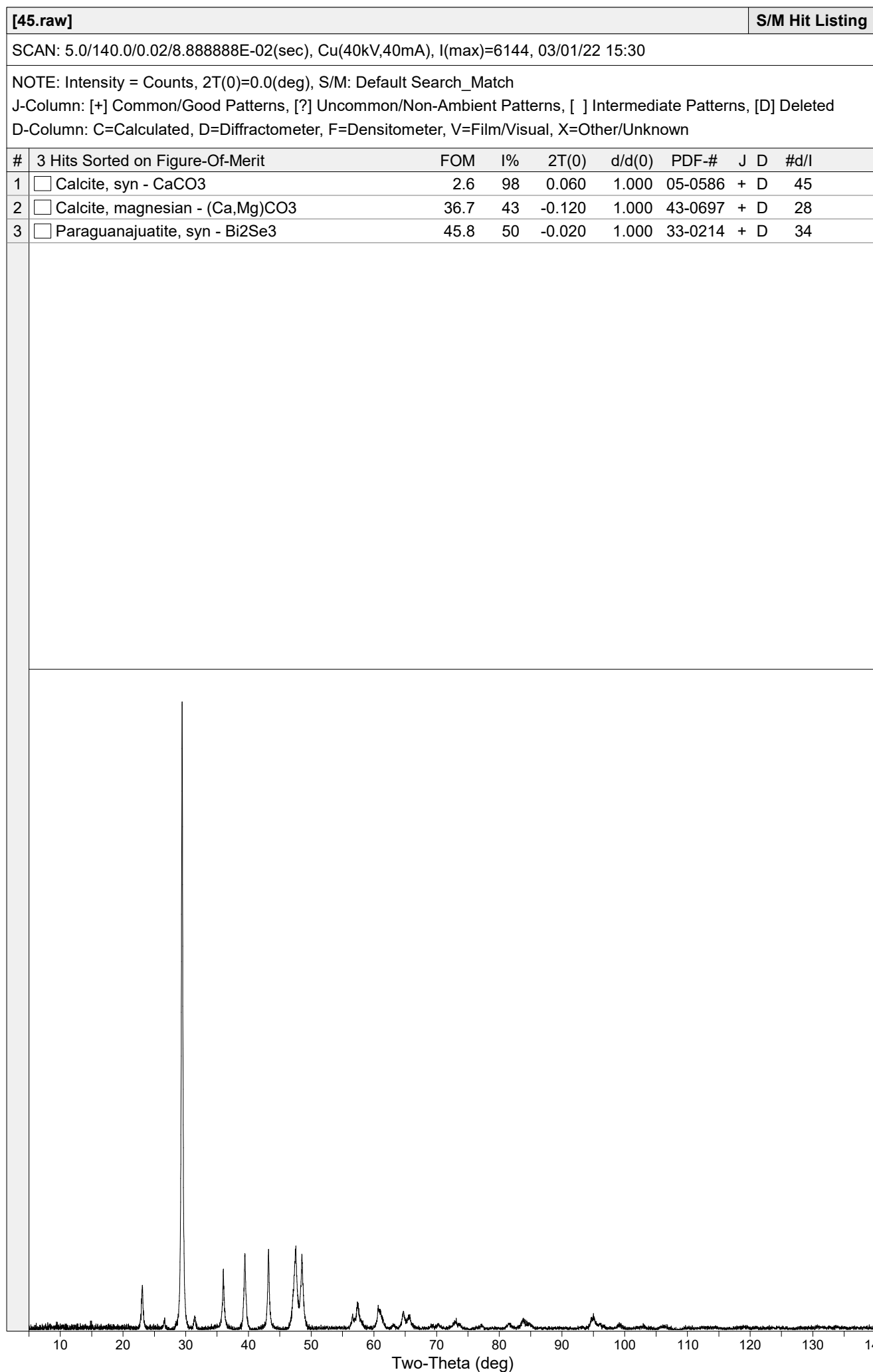

Supplement: Supplemental Information 3 [file peerj-10-13663-s003.zip › XRD Data/BZ-17.pdf]

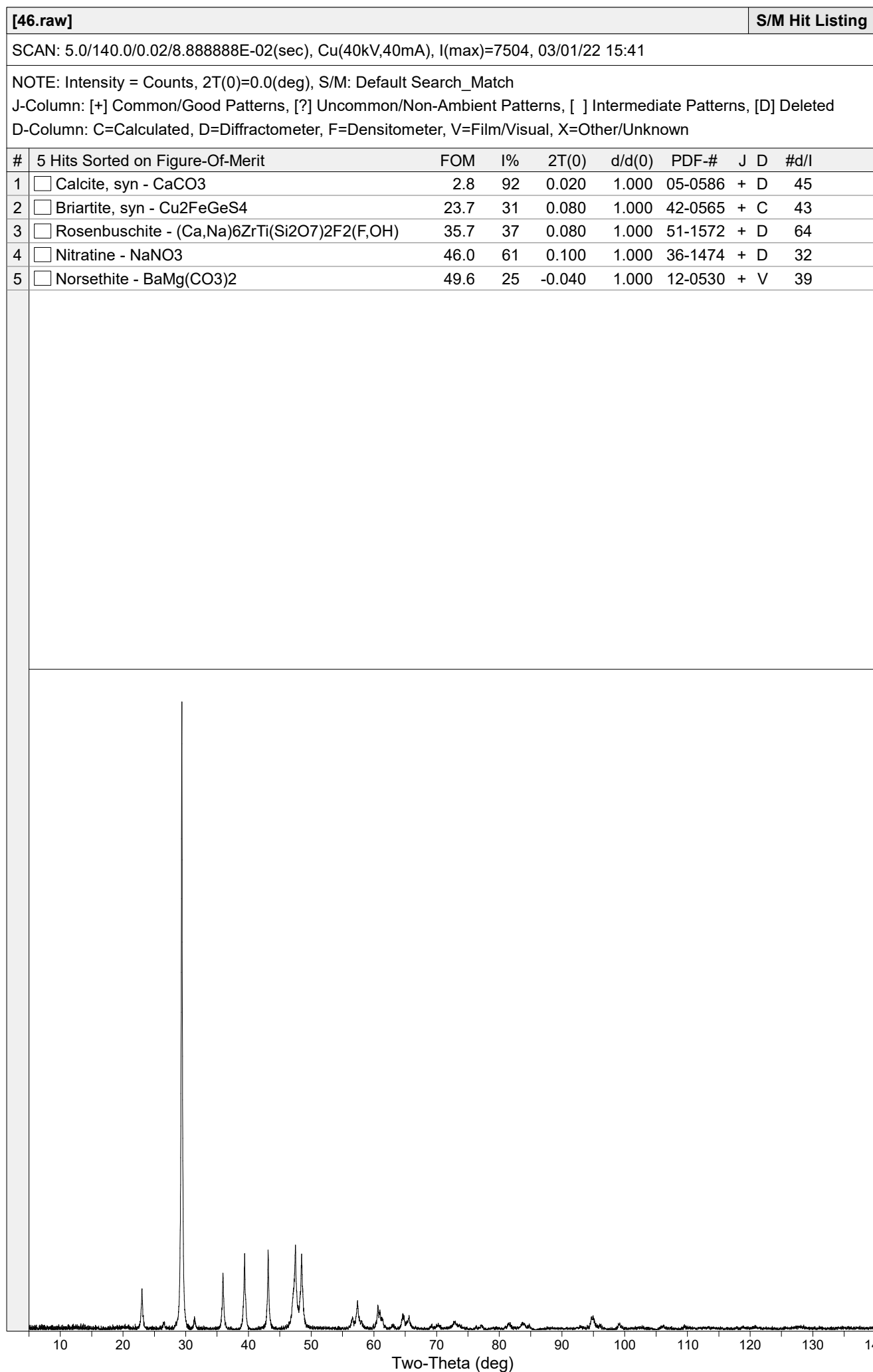

Supplement: Supplemental Information 3 [file peerj-10-13663-s003.zip › XRD Data/BZ-18.pdf]

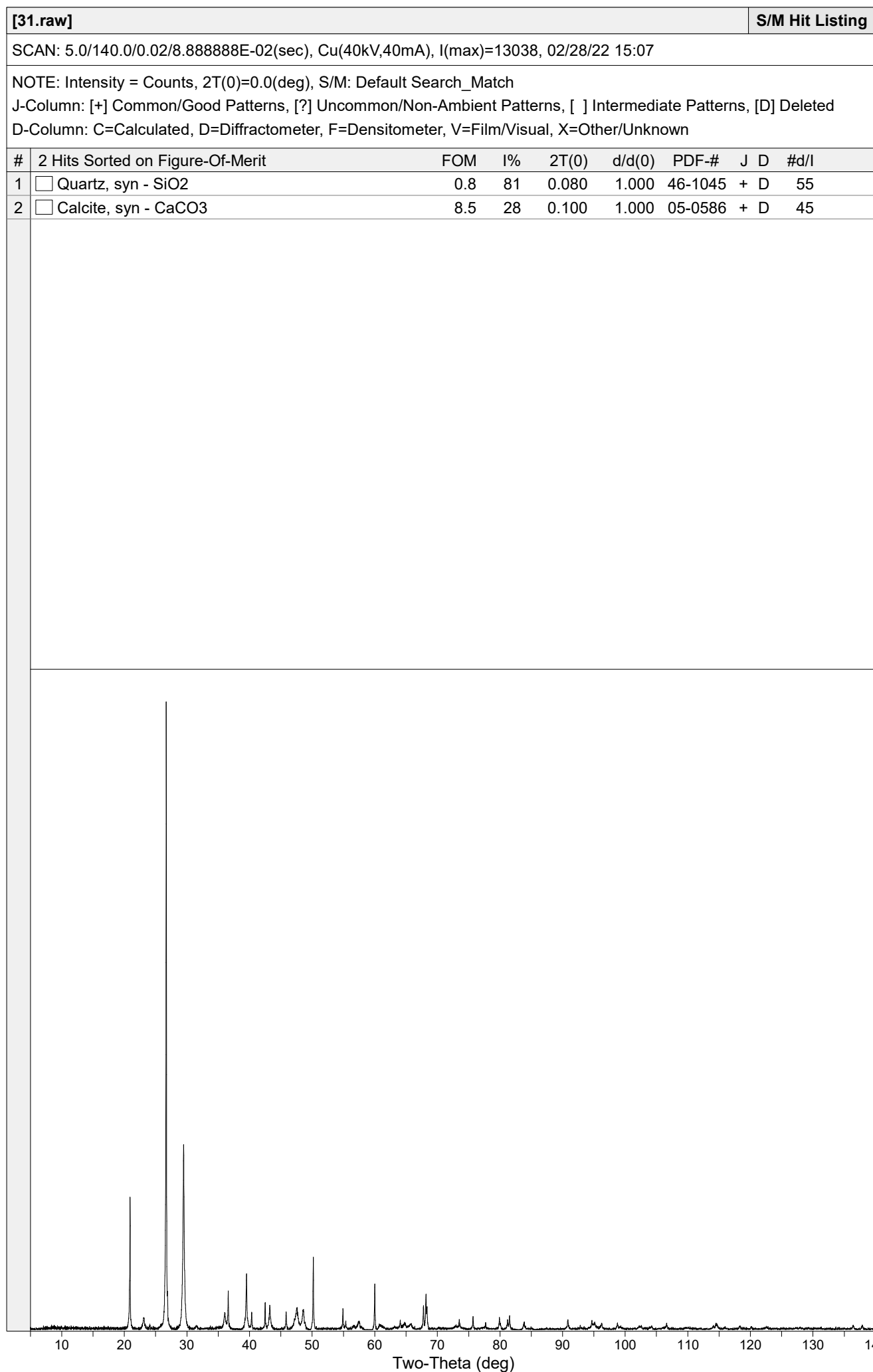

Supplement: Supplemental Information 3 [file peerj-10-13663-s003.zip › XRD Data/BZ-3.pdf]

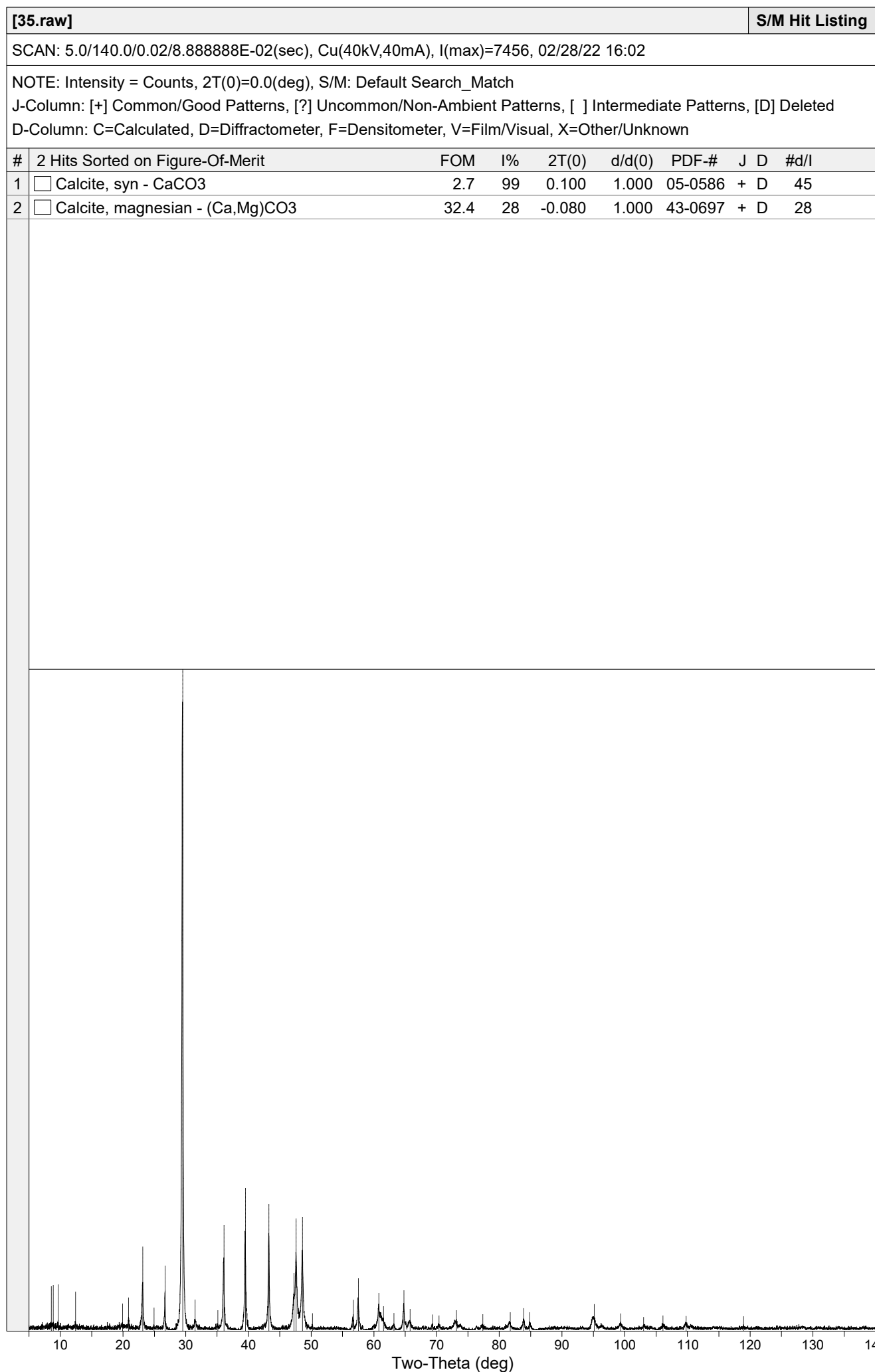

Supplement: Supplemental Information 3 [file peerj-10-13663-s003.zip › XRD Data/BZ-7.pdf]

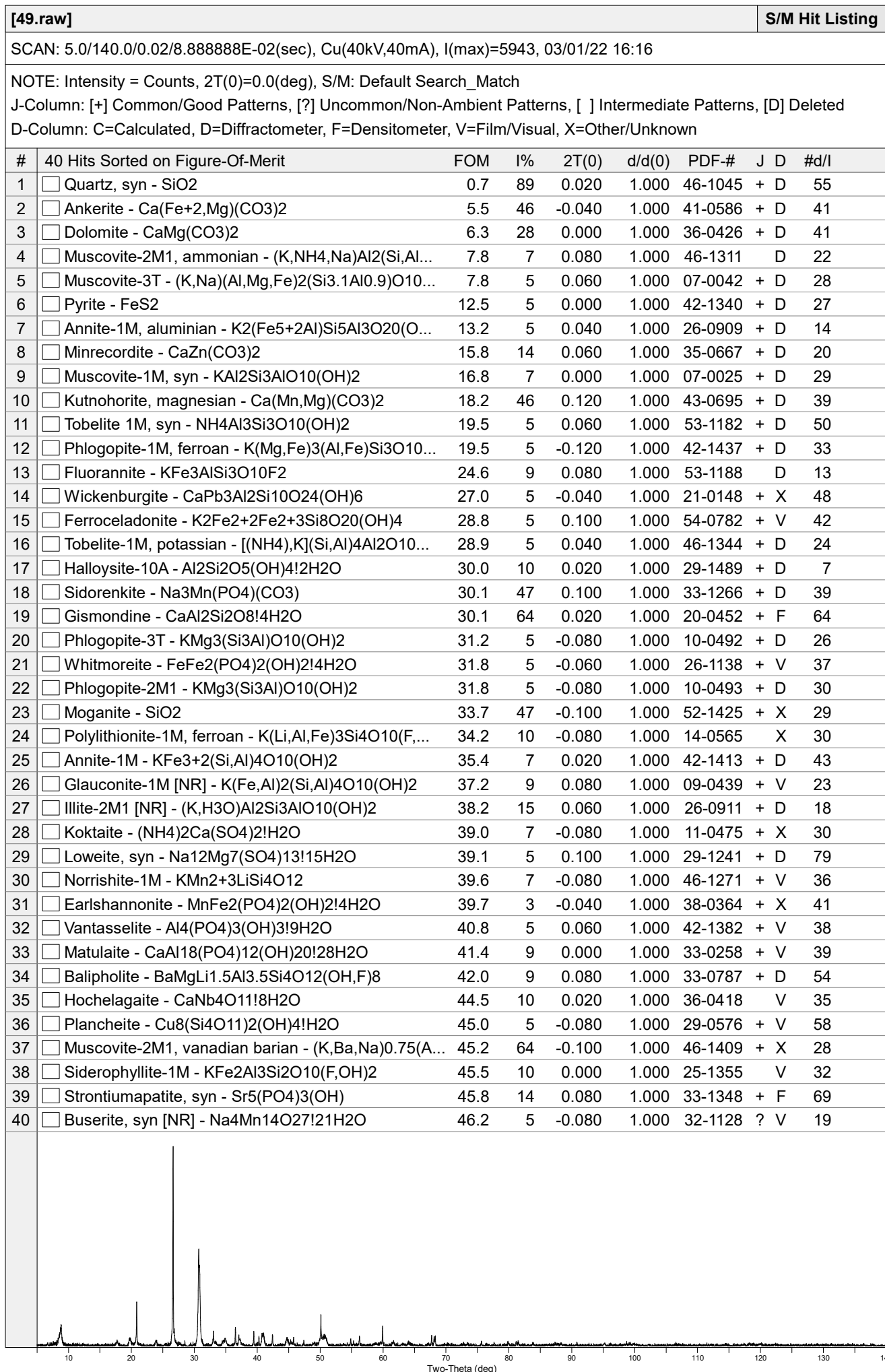

Supplement: Supplemental Information 3 [file peerj-10-13663-s003.zip › XRD Data/JWZ-1.pdf]

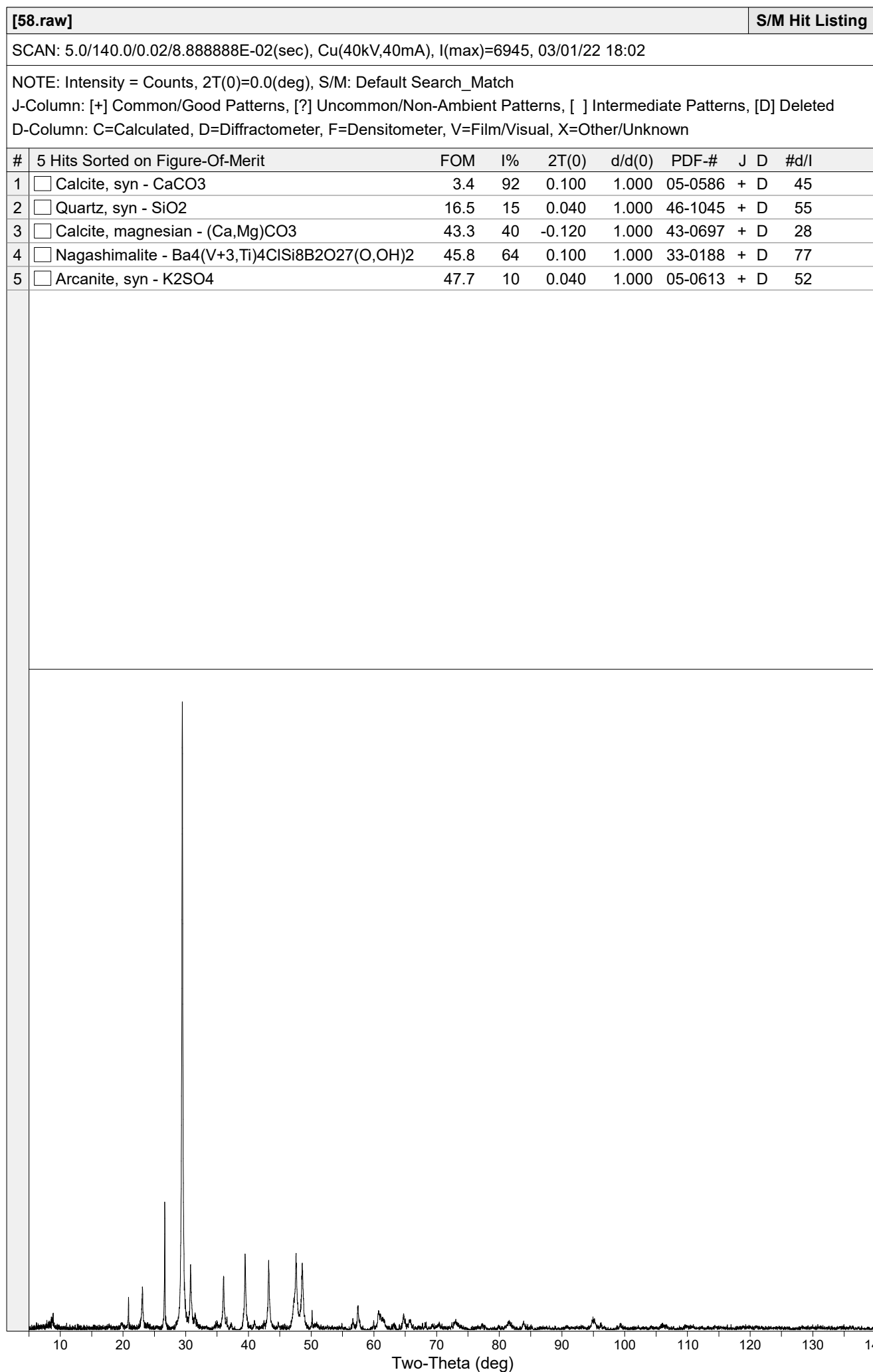

Supplement: Supplemental Information 3 [file peerj-10-13663-s003.zip › XRD Data/JWZ-10.pdf]

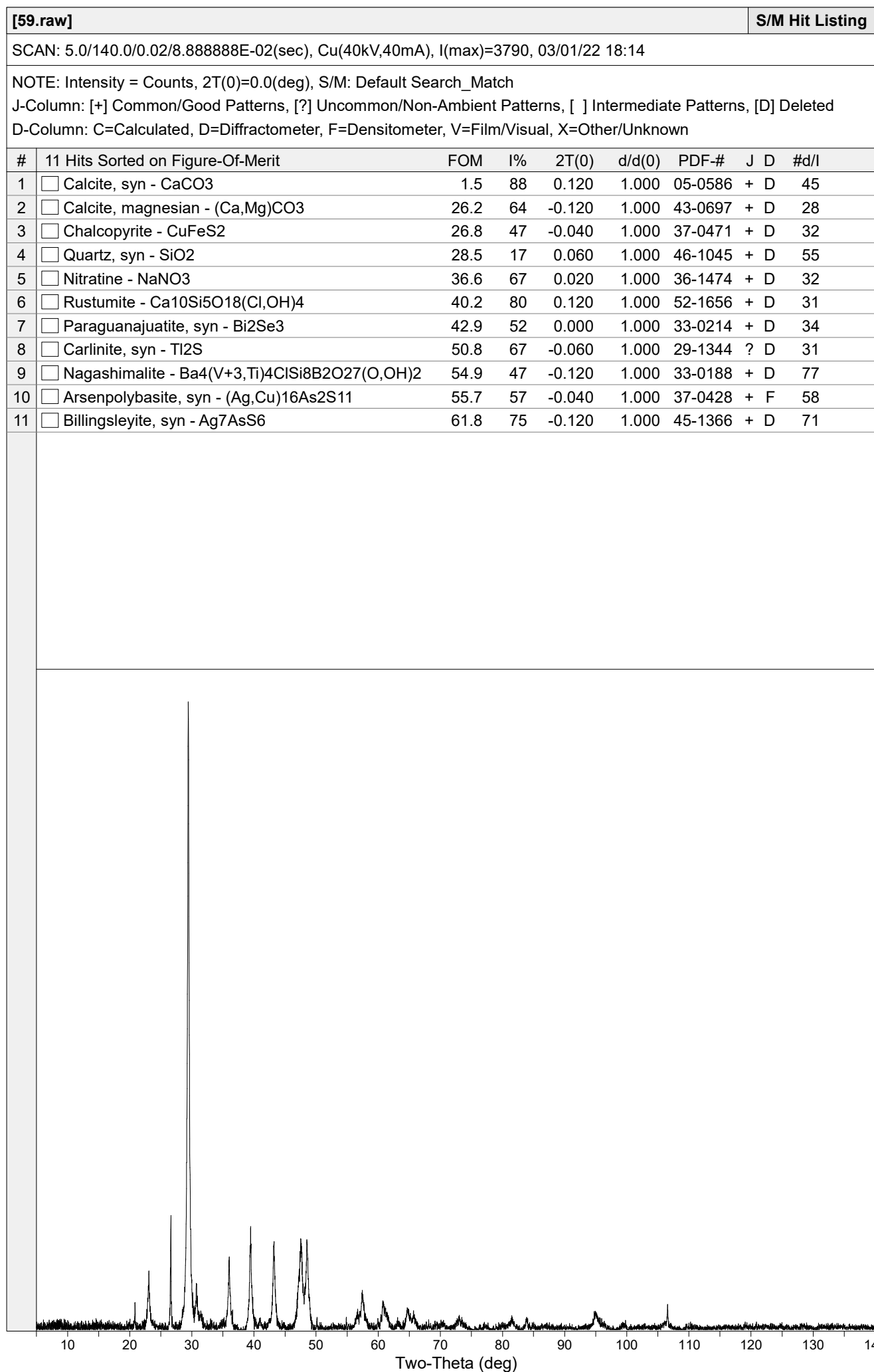

Supplement: Supplemental Information 3 [file peerj-10-13663-s003.zip › XRD Data/JWZ-11.pdf]

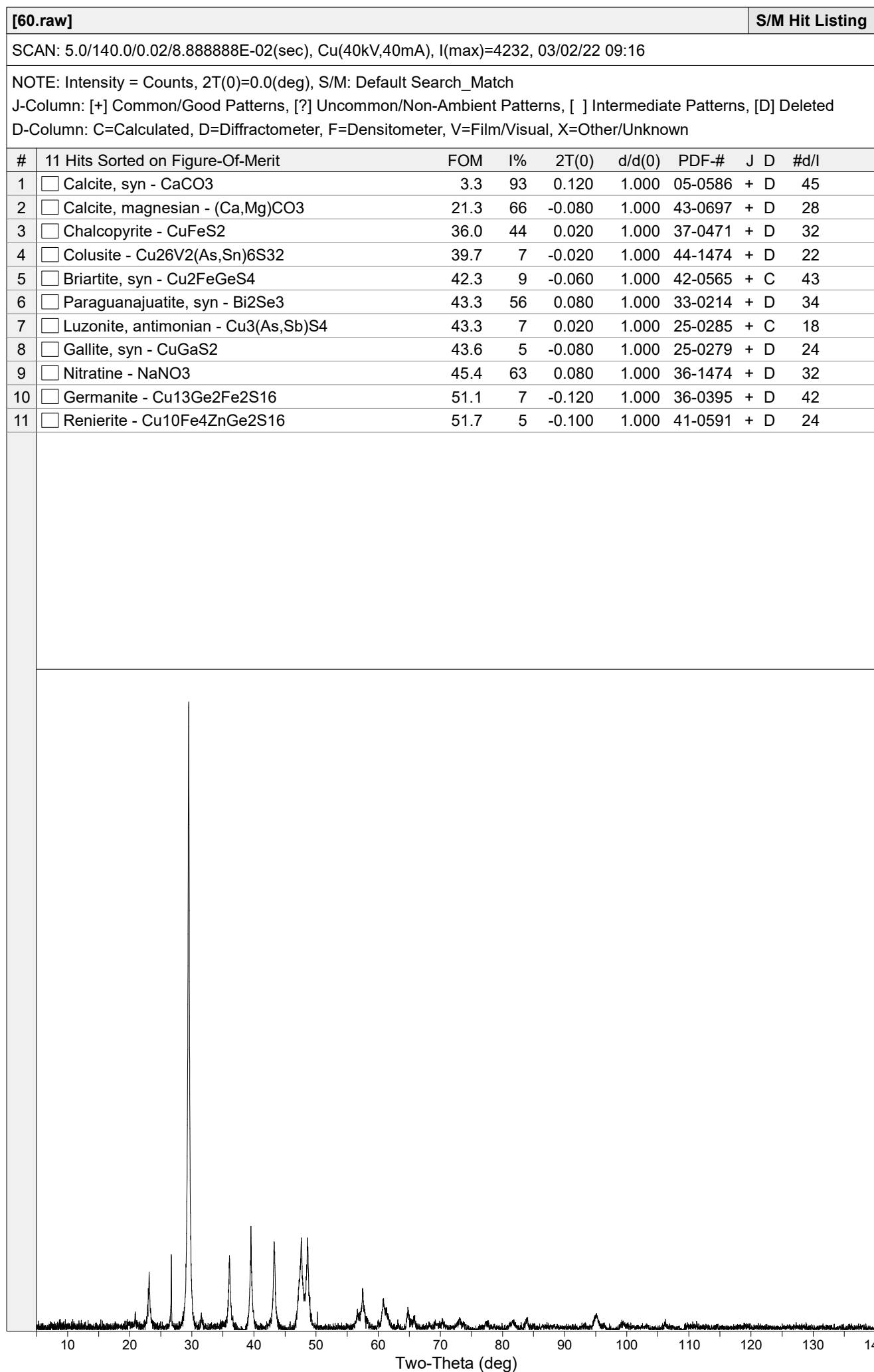

Supplement: Supplemental Information 3 [file peerj-10-13663-s003.zip › XRD Data/JWZ-12.pdf]

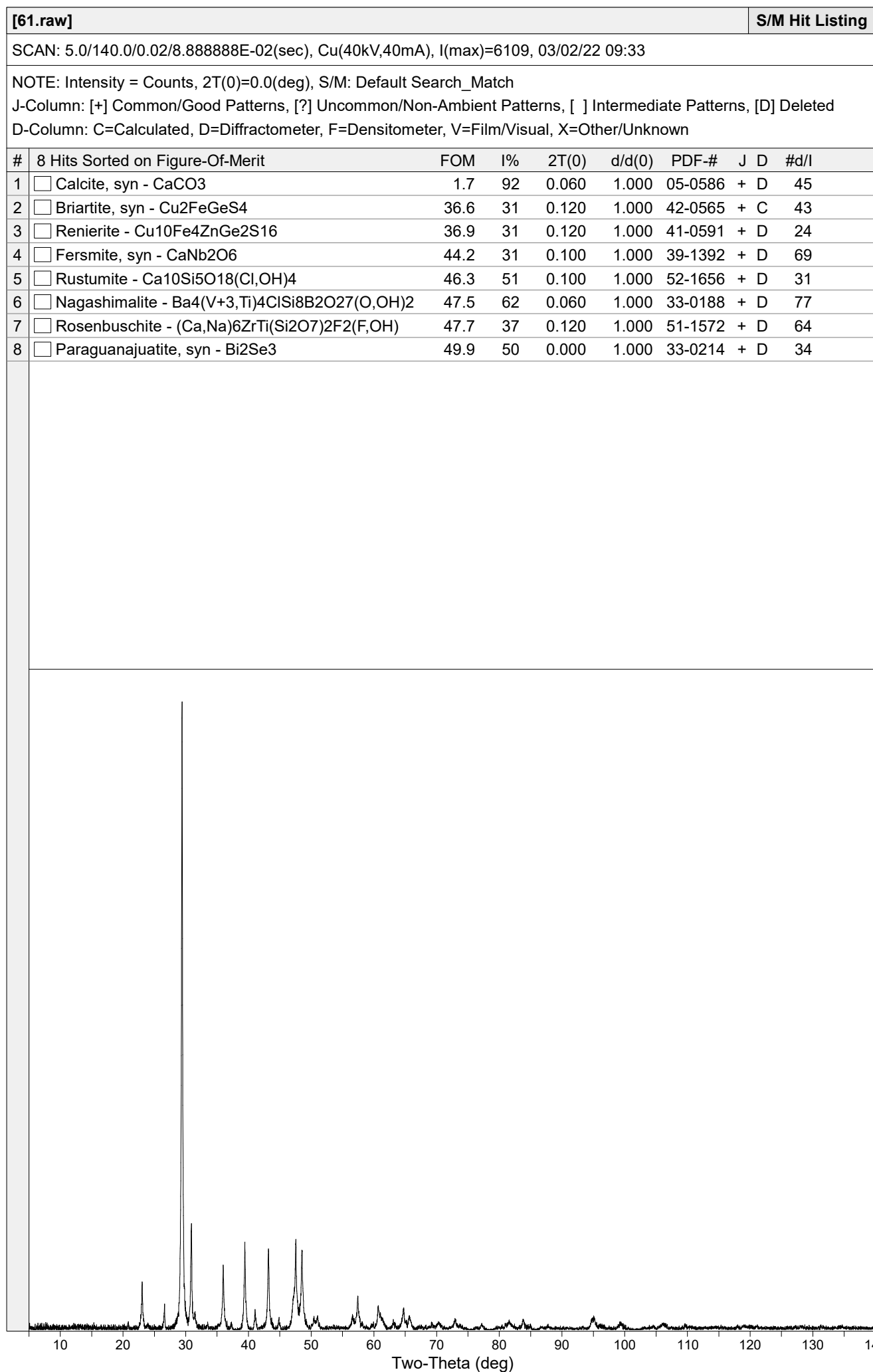

Supplement: Supplemental Information 3 [file peerj-10-13663-s003.zip › XRD Data/JWZ-13.pdf]

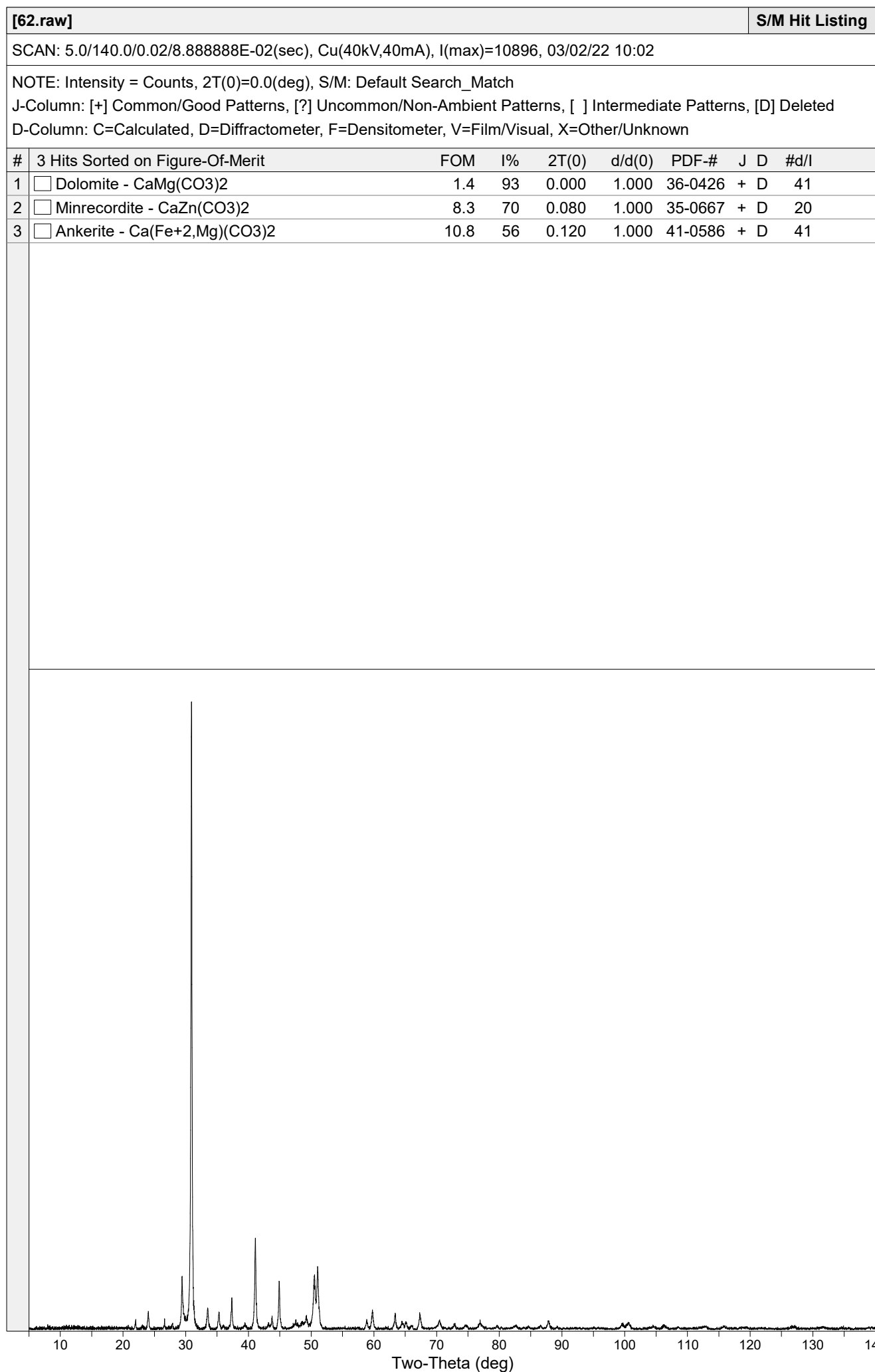

Supplement: Supplemental Information 3 [file peerj-10-13663-s003.zip › XRD Data/JWZ-14.pdf]

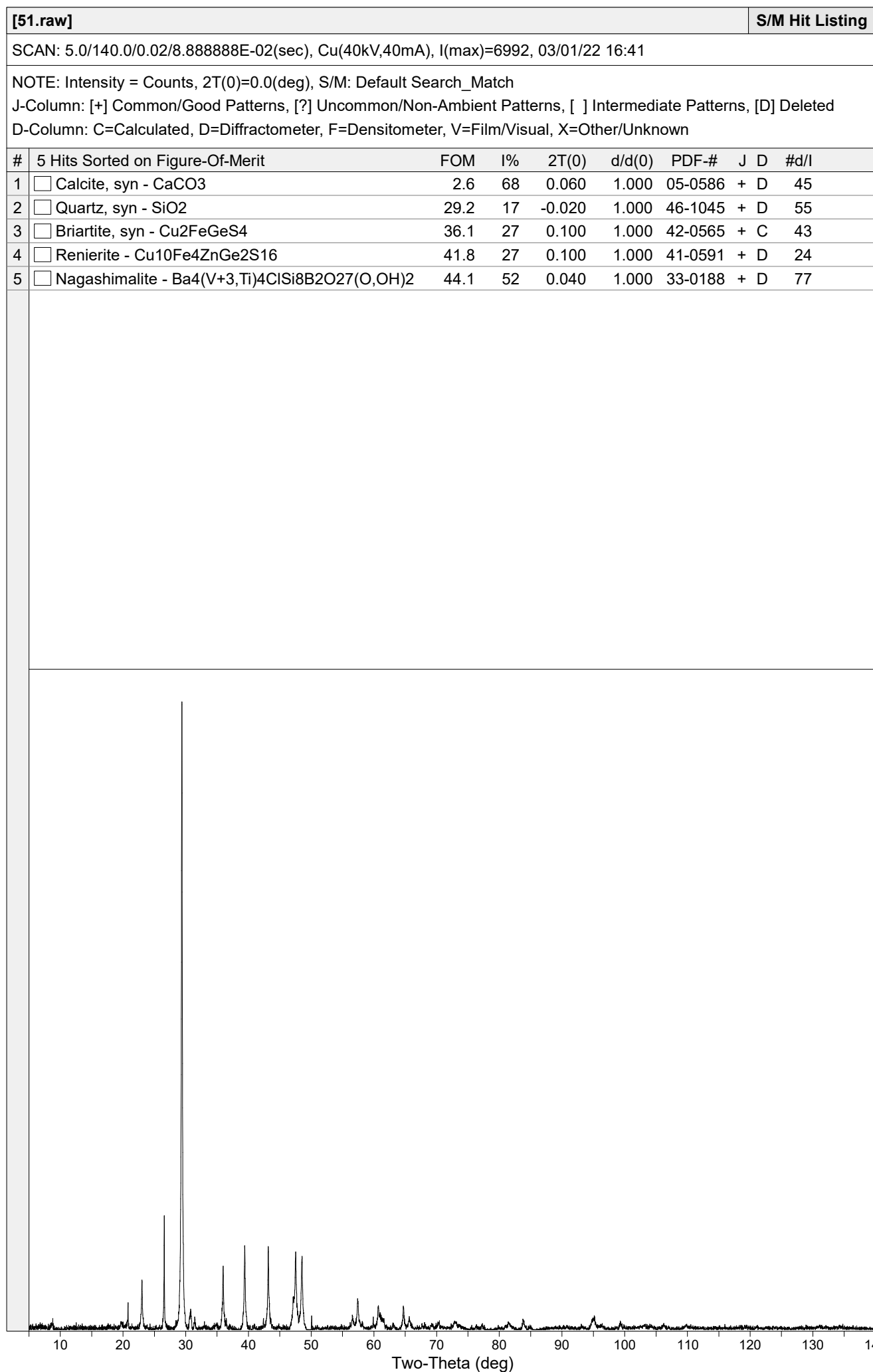

Supplement: Supplemental Information 3 [file peerj-10-13663-s003.zip › XRD Data/JWZ-3.pdf]

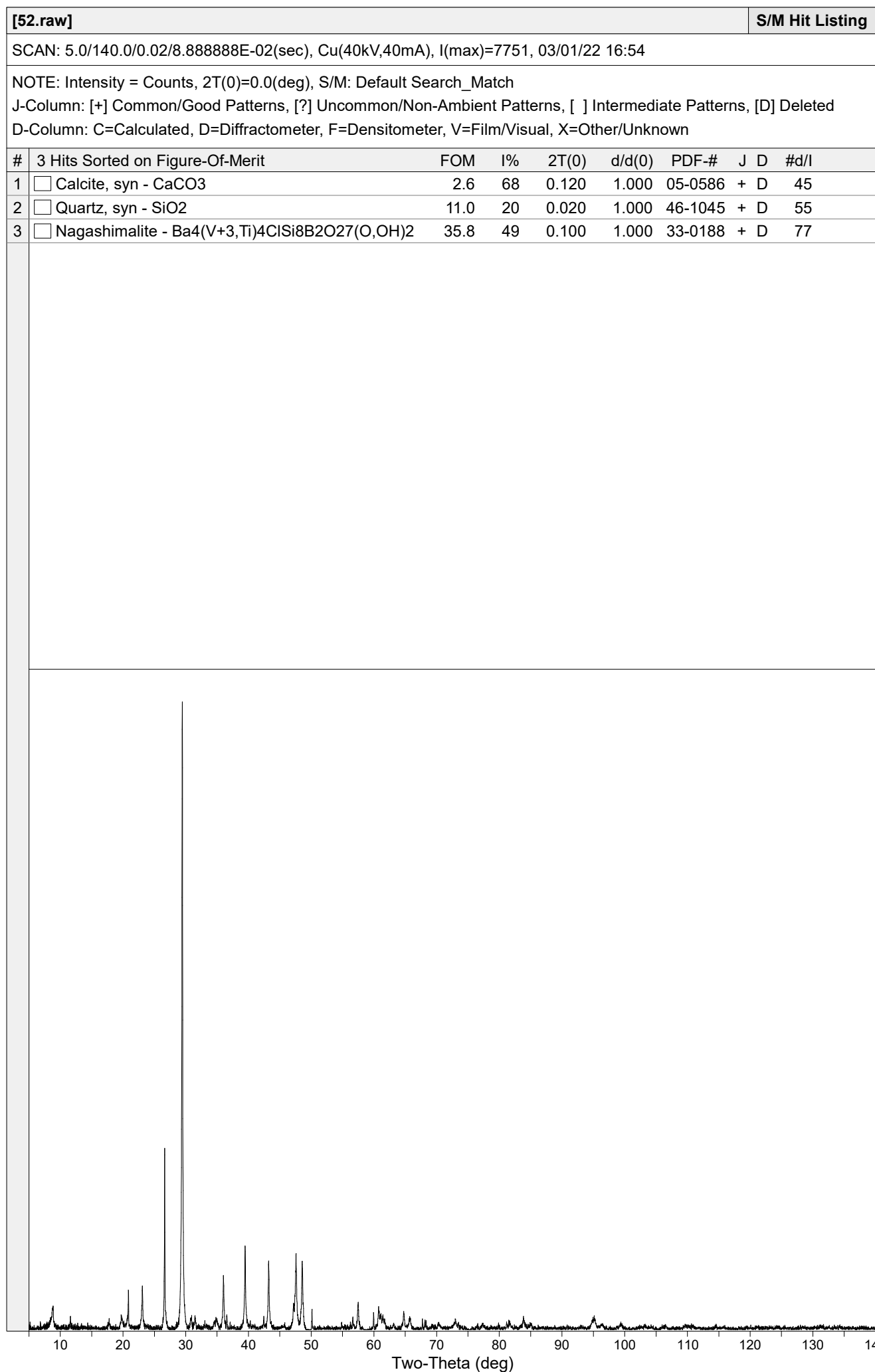

Supplement: Supplemental Information 3 [file peerj-10-13663-s003.zip › XRD Data/JWZ-4.pdf]

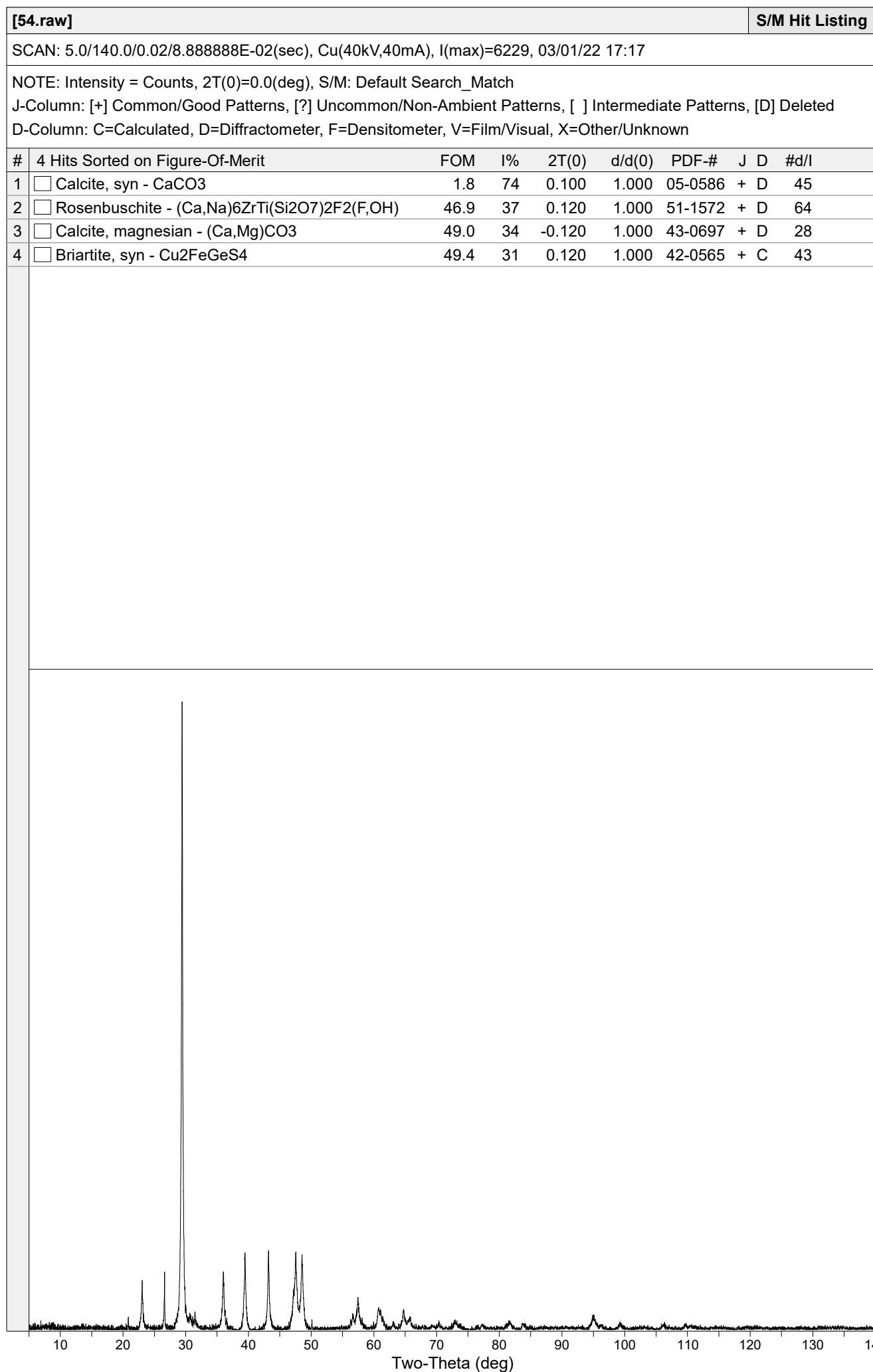

Supplement: Supplemental Information 3 [file peerj-10-13663-s003.zip › XRD Data/JWZ-6.pdf]

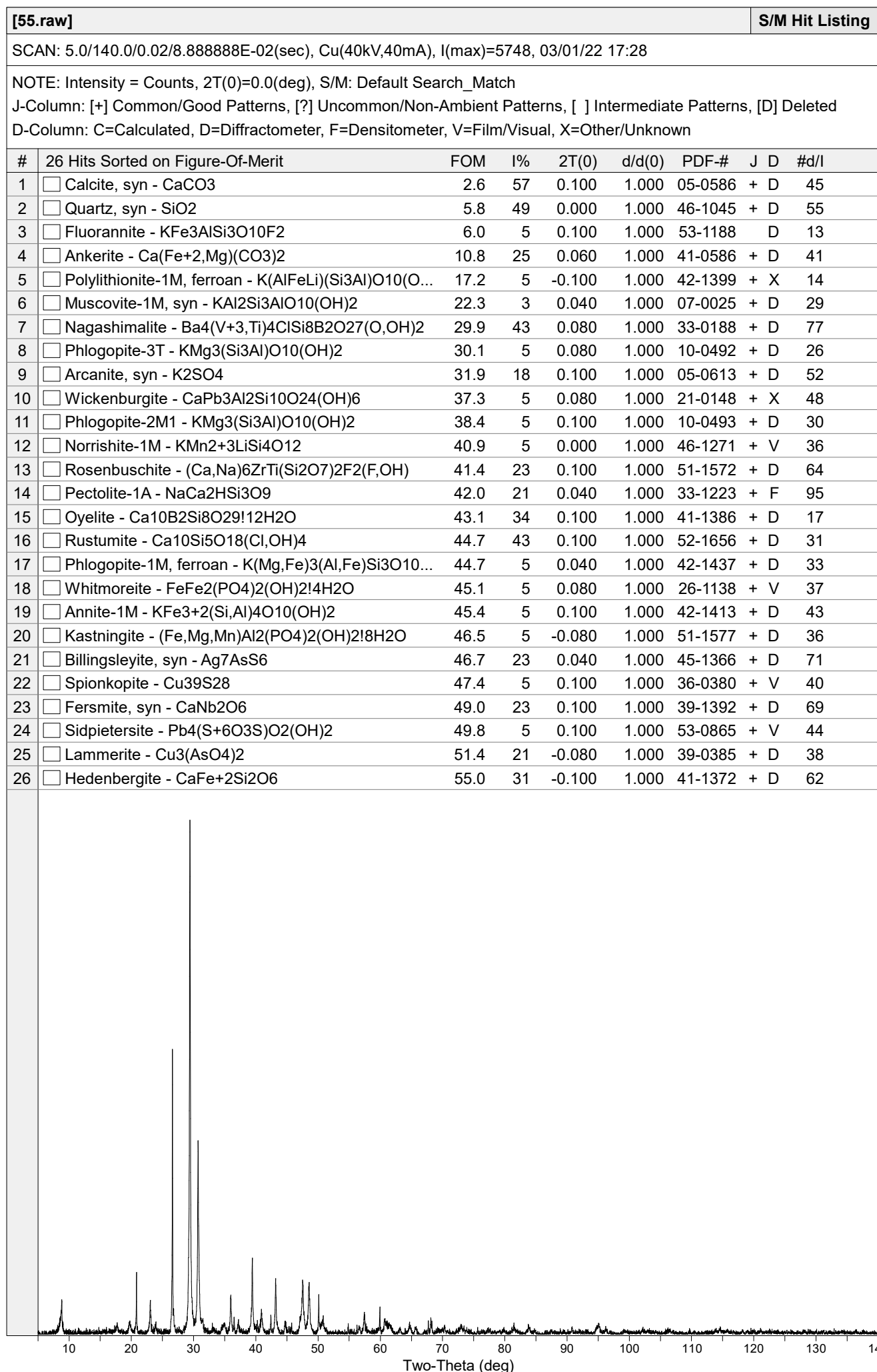

Supplement: Supplemental Information 3 [file peerj-10-13663-s003.zip › XRD Data/JWZ-7.pdf]

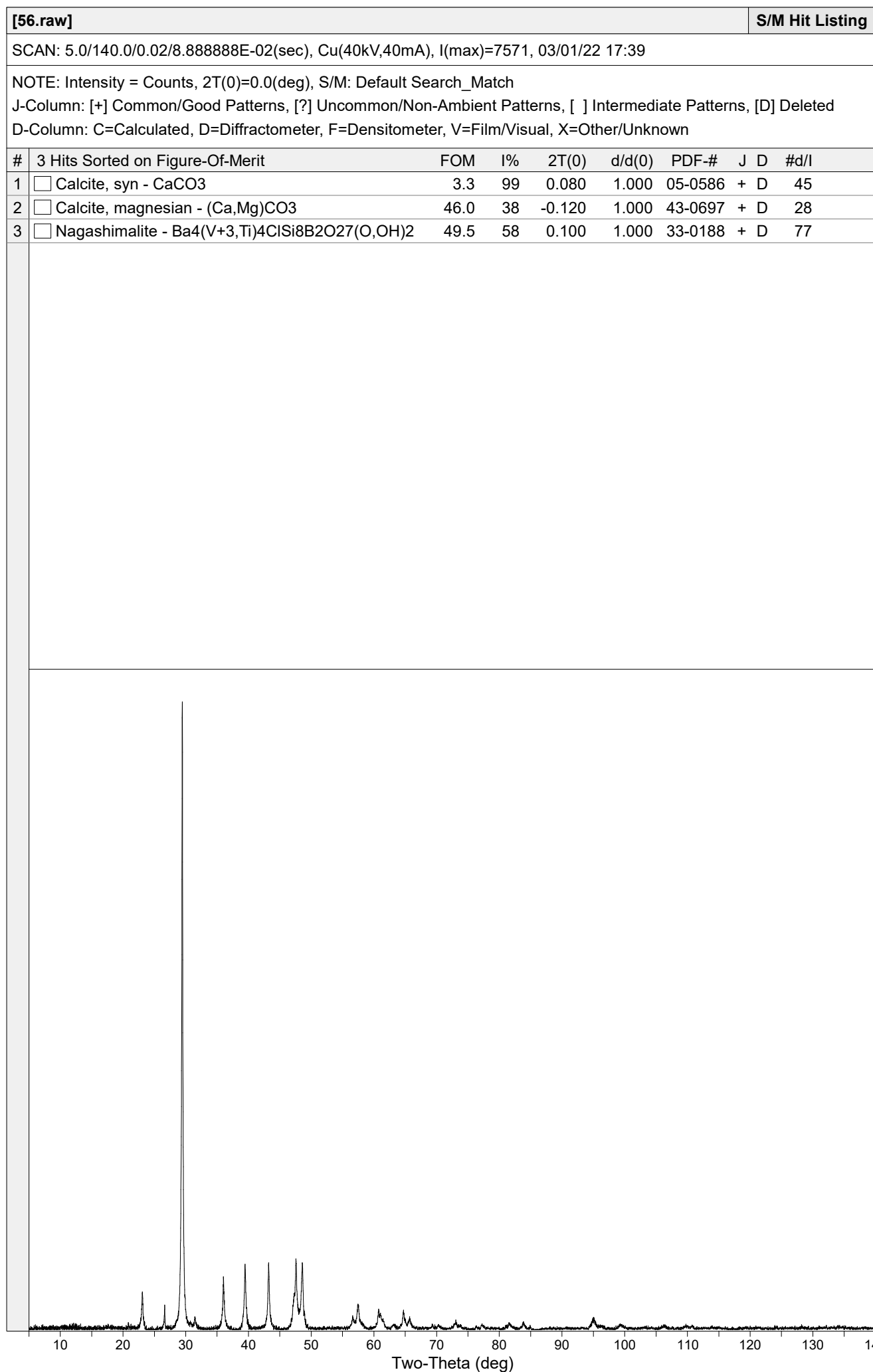

Supplement: Supplemental Information 3 [file peerj-10-13663-s003.zip › XRD Data/JWZ-8.pdf]

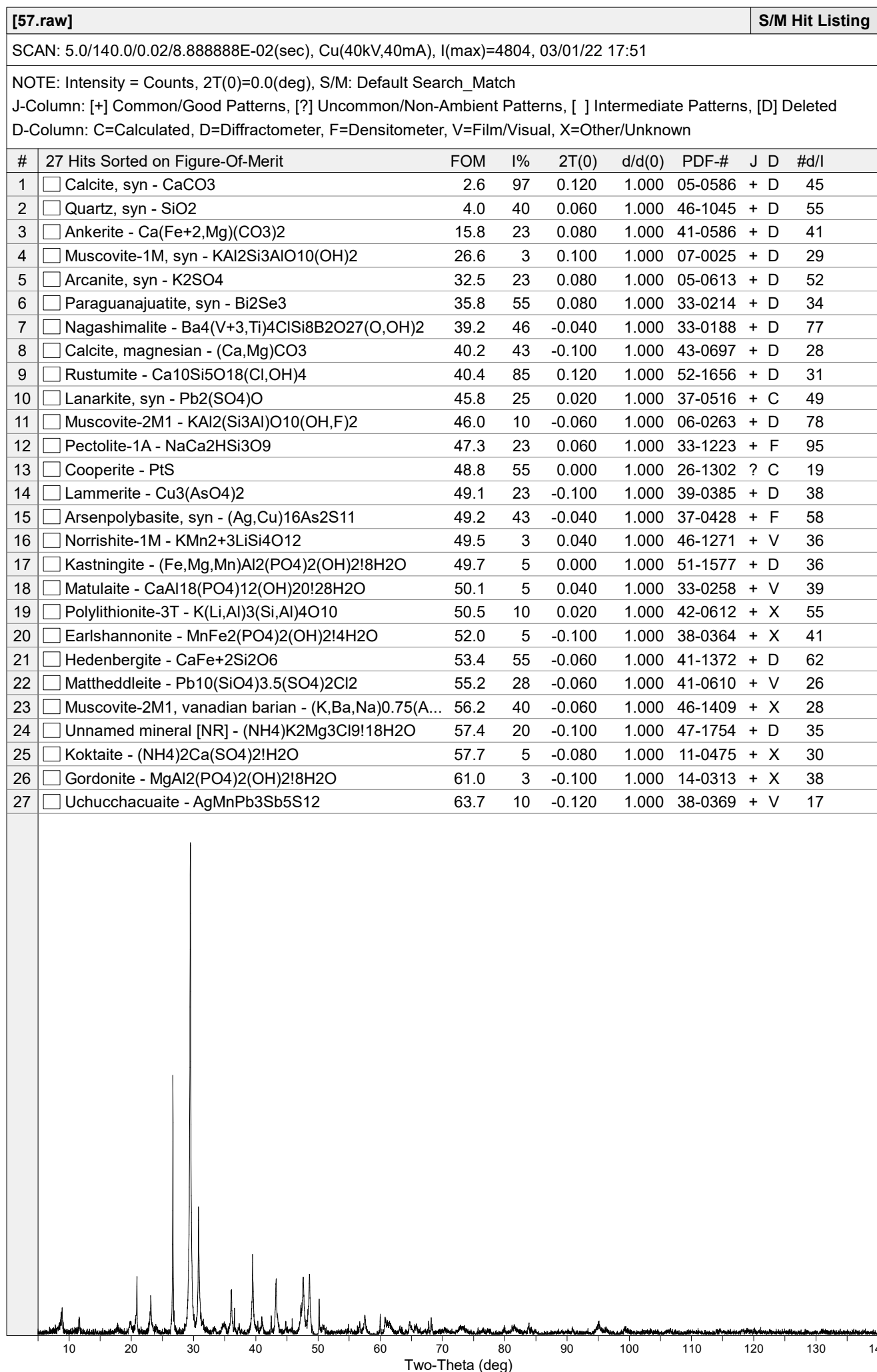

Supplement: Supplemental Information 3 [file peerj-10-13663-s003.zip › XRD Data/JWZ-9.pdf]
